# Nanoparticles in Antibacterial Therapy: A Systematic Review of Enhanced Efficacy against Intracellular Bacteria

**DOI:** 10.1021/acsomega.5c01813

**Published:** 2025-04-25

**Authors:** Pablo Mendez-Pfeiffer, Manuel G. Ballesteros Monrreal, Mayra A. Mendez-Encinas, Dora Valencia, Bryan Ortiz, Oscar González-Davis, Ruben D. Cadena-Nava

**Affiliations:** † Departamento de Bionanotecnología, Centro de Nanociencias y Nanotecnología, Universidad Nacional Autónoma de México, Km 107 Carretera Tijuana-Ensenada, Ensenada, Baja California 22860, México; ‡ Departamento de Ciencias Químico Biológicas y Agropecuarias, Universidad de Sonora, Campus Caborca, Caborca 83600, Sonora, México; § Instituto de Investigaciones en Microbiología, Facultad de Ciencias, Universidad Nacional Autónoma de Honduras, Tegucigalpa 11101, Honduras

## Abstract

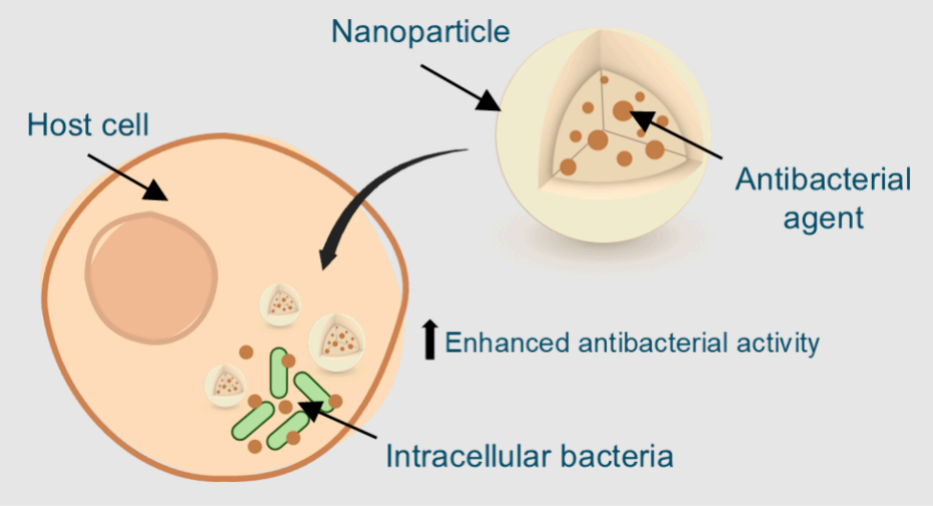

Intracellular bacterial infections represent a considerable therapeutic challenge due to the ability of pathogens to invade and replicate within host cells, hampering the action of the immune system and the effectiveness of conventional antibiotics. Bacteria such as *Mycobacterium tuberculosis*, *Listeria monocytogenes*, and methicillin-resistant *Staphylococcus aureus* (MRSA), among others, can persist within host cells, allowing them to evade the immune response and develop resistance to antibacterial treatments. A key factor in the persistence of these infections is the ability of bacteria to enter a dormant state, which reduces their susceptibility to antibiotics that affect the dividing cells. Nanotechnology is emerging as a promising solution as nanoparticle-based systems can improve the intracellular penetration of antibiotics, allow their controlled release, and reduce side effects. This review covers the development and efficacy of nanoparticle-encapsulated antibiotics in models of intracellular infections, highlighting the need to further investigate their potential to overcome the barriers of conventional therapies and improve the treatment of these complex infections.

## Introduction

Intracellular bacteria are a subset of pathogenic microorganisms that invade and replicate within the host cells. These bacteria, including facultative intracellular bacteria such as *Mycobacterium tuberculosis*, *Listeria monocytogenes*, *Salmonella typhimurium*, *Staphylococcus aureus*, *Escherichia coli*, and obligate intracellular bacteria such as *Chlamydia trachomatis* and *Rickettsia* spp., pose a significant challenge to the host’s immune system and present a formidable obstacle in clinical treatment. Their ability to reside within host cells like macrophages and epithelial cells not only shields them from the host’s immune defenses but also from many conventional antibiotics that are less effective at crossing cellular membranes.^1,2^

Understanding and effectively treating intracellular bacterial infections cannot be overstated. These infections can lead to chronic conditions and severe health complications, contributing significantly to global morbidity and mortality. The ability of intracellular bacteria to cause persistent and recurrent infections complicates treatment regimens and leads to prolonged patient suffering and increased healthcare costs.^3^

One of the primary challenges in treating intracellular bacterial infections is antibiotic resistance. Intracellular pathogens are adept at evading traditional antibiotics, either by residing within cells where antibiotics cannot easily reach them or by developing mechanisms that degrade or expel the antibiotics.^4,5^ The rise of antibiotic-resistant strains, such as methicillin-resistant *S. aureus* (MRSA), exacerbates this problem, rendering many existing treatments ineffective. Even today, with growing research focused on the discovery and development of new antibacterial agents including natural products, synthetic and chemically modified compounds, their therapeutic administration remains challenging as they may suffer from the same limitations as conventional antibiotics.^6,7^

Intracellular bacteria employ various virulence factors to penetrate and invade host cells. For example, *L. monocytogenes* uses internalins proteins to facilitate entry into epithelial cells,^8^ while *S. typhimurium* employs a type III secretion system to inject effector proteins that manipulate host cell functions, promoting bacterial uptake.^9^*E. coli*, particularly pathogenic strains like uropathogenic *E. coli* (UPEC), uses adhesins such as pili and fimbriae to attach to and invade epithelial cells.^10^ Obligate intracellular bacteria such as *C. trachomatis* and *Rickettsia* spp. use specialized secretion systems to enter host cells and create niches for replication.^11^ These virulence factors allow bacteria to hijack host cellular processes, evade immune responses, and establish intracellular niches where they can replicate and persist.

Moreover, intracellular bacteria can enter a dormant state, making them less susceptible to antibiotics that target actively dividing cells. This persistence is a significant factor in recurrent infections, where the bacteria re-emerge after the completion of antibiotic therapy. Current therapeutic strategies often fail to completely eradicate the infection, leading to cycles of remission and relapse.^3,12^

Nanotechnology offers a promising solution by enabling advanced drug delivery systems that enhance the stability, intracellular penetration, and effectiveness of antibacterial agents.^13,14^ Functionalized nanoparticles can target infected cells precisely, ensuring higher intracellular concentrations while minimizing off-target effects and reducing the required therapeutic doses. Additionally, controlled and sustained drug release helps address bacterial persistence and recurrence.^15^

Furthermore, nanoparticles can encapsulate multiple therapeutic agents, allowing for a combination therapy within a single delivery system. This approach can enhance antibacterial efficacy through synergistic mechanisms, reduce the likelihood of resistance development, and simultaneously target multiple bacterial survival pathways.^16^

Despite the critical importance of intracellular infections to the global health sector and the growing advancements in drug delivery technologies, there remains a scarcity of studies dedicated to enhancing the efficacy of antibacterial agents using these innovative approaches. This gap underscores the need to explore how nanoparticulate systems can address the challenges posed by intracellular pathogens and improve therapeutic outcomes.

This systematic review focuses on reports of the development of antibacterial agents loaded into nanoparticles, highlighting the most studied intracellular bacterial models, the most commonly employed nanoparticulate systems, as well as their physicochemical characteristics, which enhance the effects of conventional and nonconventional antibacterial agents. This allows evaluation of whether the encapsulation of these agents into nanoparticle systems increases their efficacy and could be implemented as new formulations for the treatment of infections caused by intracellular bacteria.

## Materials and Methods

A search for published research articles was conducted in three databases: PubMed, Scopus, and Web of Science. The search strategy included terms allowing the identification of articles related to the use of antibacterial agents encapsulated in nanoparticles in order to evaluate their capacity to eradicate intracellular bacteria. The terms implemented for the search in each database were:

PubMed: ((nanotechnology OR nanoparticles OR drug delivery systems) AND (“intracellular bacteria”)) AND (combat OR treatment OR therapy OR eradicate OR eliminate).

Scopus: TITLE-ABS-KEY (nanotechnology OR nanoparticles OR drug AND delivery AND systems) AND TITLE-ABS-KEY (“intracellular bacteria”) AND TITLE-ABS-KEY (combat OR treatment OR therapy OR eradicate OR eliminate).

Web of Science: TS = (nanotechnology OR nanoparticles OR drug delivery systems) AND TS = (“intracellular bacteria”) AND TS = (combat OR treatment OR therapy OR eradicate OR eliminate).

The selected period was from 2014 to 2024, and data was recorded in a Microsoft Excel database for the evaluation of inclusion and exclusion criteria.

### Inclusion and Exclusion Criteria

#### Inclusion Criteria

Original research articles on nanoparticles’ use against intracellular bacterial infections. Studies involving human subjects, animal models, or *in vitro* models infected with intracellular bacteria. Research reporting outcomes related to bacterial clearance, load reduction, or enhanced efficacy of antibiotics and antibacterial agents in nanoparticle form. Articles written in English. Studies focused on nanoparticle size, shape, surface properties, and functionalization to enhance their therapeutic efficacy. Studies using nanoparticles for encapsulating or functionalizing antibacterial agents to improve delivery, stability, and effectiveness as compared to free agents.

#### Exclusion Criteria

All review articles, meta-analyses, case reports, and editorial letters. Articles that do not include intracellular bacterial models. Studies focused on nanomaterials used for purposes other than combating intracellular bacteria. Articles not in English. Limited access to the full article. Articles that did not compare free antibacterial agents against encapsulated antibacterial agents.

### Selection of Studies

All articles were reviewed by PMP, MGBM and BO. Duplicates were excluded based on DOI and title. All reviews, short surveys, letters, editorial materials, book chapters, and perspectives were also excluded. The remaining articles were reviewed based on title and abstract to determine relevance to the study, all research that was not relevant was excluded. Candidate articles were retrieved for full reading by PMP, OGD and RCN; those articles that could not be retrieved were excluded from the study. Selected articles were reviewed by PMP and MGBM according to inclusion and exclusion criteria. Any differences between reviewers were analyzed by DV and MME.

### Data Extraction

Data of the selected articles were classified using Microsoft Excel according to Nanoparticle formulation, type of nanoparticle, antibacterial agent, bacterial model, cell model, *in vivo* model, nanoparticle size, nanoparticle zeta potential, encapsulation efficiency/drug loading, antibacterial efficacy, and reference.

## Results

**Figure 1:**
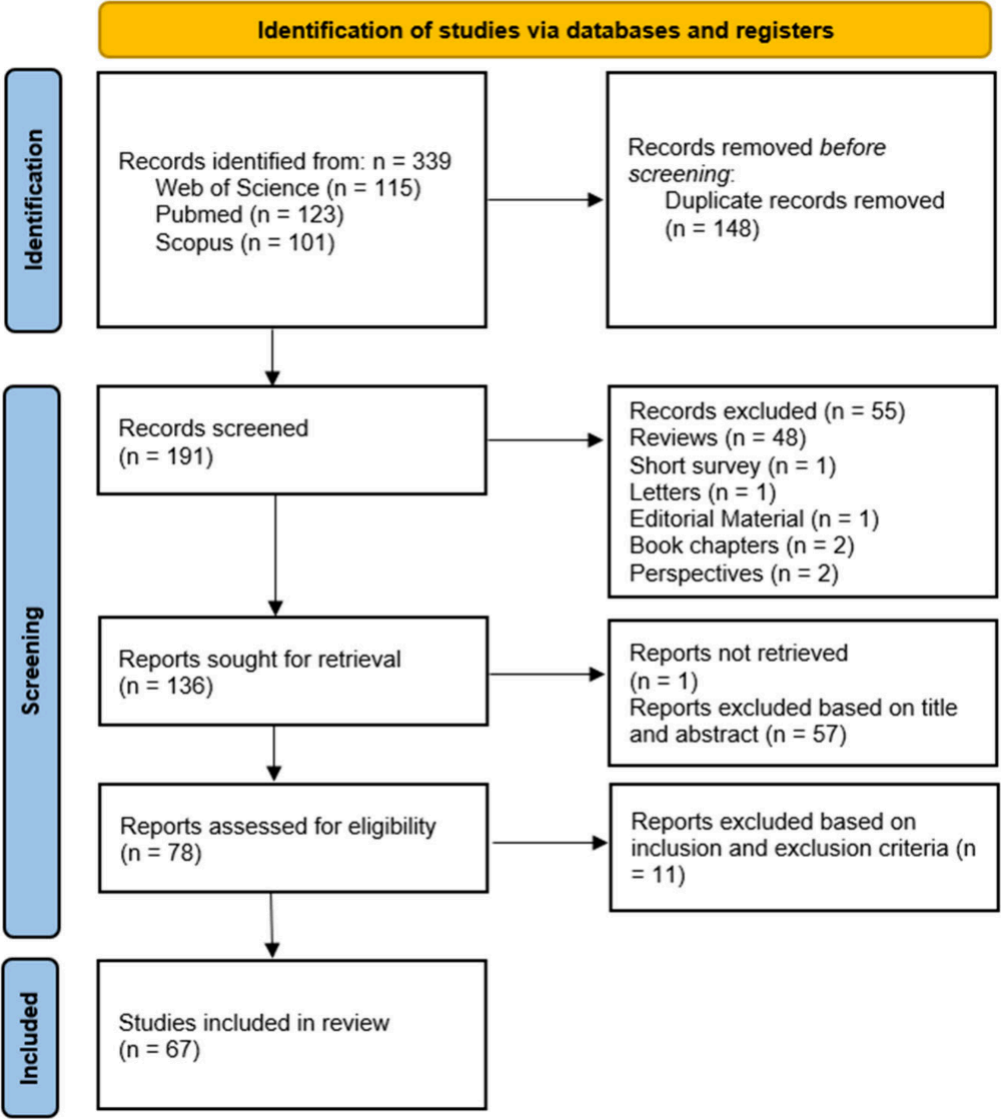
PRISMA flowchart for the literature search and study selection.

A total of 339 research articles were identified, including 115 from Web of Science, 101 from Scopus, and 123 from PubMed. After duplicates were removed (*n* = 148), 191 unique records remained. Subsequently, reviews (*n* = 48), short surveys (*n* = 1), letters (*n* = 1), editorial material (*n* = 1), book chapters (*n* = 2), and perspectives (*n* = 2) were excluded. A total of 136 records were screened based on titles and abstracts, leading to the exclusion of 57 records. One record was not retrievable. Finally, 78 records underwent full-text evaluation, of which 67 met the inclusion criteria (Figure 1). Finally, data extracted from these 67 studies was organized in Table 1.

**Table 1:** Type of Nanoparticles Used for the Delivery of Antibacterial Agents, Physicochemical Characteristics, and Antibacterial Efficacy

Nanoparticle Composition	Nanoparticle Type	Antibacterial Agent	Bacterial Model	Cell Line Model	Animal Model	Size (nm)	Zeta Potential (Mv)	Encapsulation Efficiency/Drug Loading (%)	Reported Intracellular Antibacterial Efficacy	ref.
Oxidized dextran (ox-Dex) and selenocystamine dihydrochloride encapsulating vancomycin (Van)	Polymeric	Vancomycin	*Staphylococcus aureus*	RAW 264.7 Macrophages	Balb/c rats	35	Not specified	–/11.8	Bacterial viability (%)	(52)
Biodegradable polymeric nanoemulsions using guanidinium-functionalized poly(oxanorborneneimide) (PONI-GMT) polymers	Polymeric	Eugenol	*Staphylococcus aureus*	RAW264.7 Macrophages	Balb/c mice	120	25	Not specified	Log CFU/mL: E-BNE > Eugenol > PONI-GMT	(53)
Cyclic RGD peptide functionalized liposomes (cRGD-sLip)	Lipidic	Vancomycin	*Staphylococcus aureus*	Macrophages	ICR mice	120	50	–/4.69	MRSA Counts (CFU): cRGD-sLIP/Van > sLip/Van > Free Van	(54)
Hyaluronic acid-cholesterol nanohydrogels (HA-CH NHs) loaded with levofloxacin (LVF)	Hybrid	Levofloxacin	*Staphylococcus aureus and Pseudomonas aeruginosa*	HeLa cervical cells	–	170 ± 10 to 195 ± 15 nm	Not specified	–/5.0 ± 0.5	Intracellular CFUs: LVF-Nh freeze-dried > LVF-NH > LVF free	(55)
Poly(lactic-*co*-glycolic acid) (PLGA) microparticles	Polymeric	Nitrofurantoin	*Enterococcus faecalis*	HBLAK bladder cells	–	2800	Not specified	94/10	Median CFU/mL and 95% CI: CapFuran 2 mg/mL > Free Nitrofurantoin > Capfuran Placebo	(56)
Gentamicin-conjugated vanadium oxide nanoparticles	Inorganic	Gentamicin	*Staphylococcus aureus*	RAW264.7 Macrophages	Balb/c mice	14.4	14.7	–/28.49	CFU/mL: GEN-NPs > Gen + NPs = NPs > GEN	(57)
AZT-liposomes (composed of Lipoid E PC S, egg phosphatidylglycerol, DODAB, Tween 80).	Hybrid	Azithromycin	*Chlamydia trachomatis*	HeLa cervical cells	–	164 ± 10 to 187 ± 20	Not specified	25 to 37/–	*C. trachomatis* genome concentration: Anionic Liposomes > Cationic Liposomes > Neutral Liposomes > Free Azithromycin	(58)
Liposomes with doxorubicin in the core and sushi S3 peptide on the surface, functionalized with folic acid	Lipidic	Sushi S3 peptide	*Salmonella typhi*	Huh-7 hepatocyte cells	–	145 ± 20	Not specified	∼90/–	Bacterial viability (%): FSDL > SDL > SL > D + S > S	(59)
Metal–organic framework (ZIF-8) with encapsulated tetracycline and hyaluronic acid surface functionalization.	Hybrid	Tetracycline	*Staphylococcus aureus and Salmonella*	RAW264.7 Macrophages	Balb/c mice	500	Not specified	59.7/–	Clearance rate (%): TZH > ZIF-8@HA > Tet@ZIF-8 > Tet > ZIF-8	(33)
Gingerol-loaded alginate-coated niosomal nanoparticles	Hybrid	Gingerol	*Staphylococcus aureus, Pseudomonas aeruginosa*	MDA-MB-231 breast cells	–	140 ± 5 to 241 ± 9	– 26.1 ± 1.1 to −15.4 ± 1.5	76.3 ± 1.3 to 91.6 ± 1.2/–	Bacterial viability (%). Nio-Gin@AL and Nio-Gin more effective compared to Gin	(48)
Mannosylated preactivated hyaluronic acid (Man-PTHA) coated self-nanoemulsifying drug delivery system (SNEDDS)	Lipidic	Ciprofloxacin	*Salmonella typhi*	RAW264.7 Macrophages	–	257.9 to 281.4	–45 to −15	45 to 95/–	Survival (%): Man-PTHA > Man > PTHA > Cip-SNEDDS > SNEDDS	(60)
MIL-100(Fe) (iron-based metal–organic framework).	Hybrid	Methylene blue (MB)	*Chlamydia trachomatis*	RAW264.7 Macrophages	–	57	Not specified	84/2	Chlamydia titer (IFU/mL): MB-loaded NanoMOFs (light) > Free MB (light) > Free MB (dark) > MB-loaded NanoMOFs (dark) > NanoMOFs (light) = NanoMOFs (dark)	(61)
Hyaluronic acid-streptomycin conjugate (HS)	Polymeric	Streptomycin	*Staphylococcus aureus, Salmonella typhimurium and Enterococcus*	RAW264.7 Macrophages	C57BL/6 mice	200–1500	–12.6 to −4.5	–/4.5	10^3^ CFU/10^5^ cells: HSLGS-S > HA + Str + Gla	(62)
Lactoferrin nanoparticles loaded with rifampicin (Rif@Lf NPs)	Protein-based	Rifampicin	*Escherichia coli, Staphylococcus aureus and Mycobacterium marinum*	RAW264.7 Macrophages	Balb/c mice	116.8 to 123.6	5.3 to −11.5	28.9/–	CFU Well (Log10): Rif@Lt NPs > Rif@BSA NPs > Rif	(63)
Poly(lactic-*co*-glycolic acid) (PLGA) loaded with SV7	Polymeric	SV7 (a novel benzophenone-based antibiotic)	*Staphylococcus aureus*	J774A.1 Macrophages	Galleria mellonella	∼253.7 ± 16.8 to ∼277.2 ± 37.1	–17	59.4 ± 2.4 to 42.8 ± 22.8/–	Fold differences: PLGA_2.5SV7 > SV7 > PLGA_0SV7	(64)
Mesoporous iron carboxylate-based nanoMOFs (MIL-100(Fe)) loaded with Amoxicillin (AMOX) and Potassium Clavulanate (CL)	Inorganic	Amoxicillin and Potassium clavulanate	*Staphylococcus aureus*	J774–1 Macrophages	–	273 ± 23 to 298 ± 9	–18 ± 3 to 22 ± 1	–/36	Bacterial load (%): AMOX + CL nanoMOF > AMOX nanoMOF > nanoMOF > Free AMOX > Free CL	(47)
Photoluminescent peptide nanoparticles (PLPNs) encapsulated with Carvacrol (CV)	Protein-based	Carvacrol	*Staphylococcus epidermidis and Escherichia coli*	RAW264.7 Macrophages and HC-11 breast cells	–	∼224 to ∼382	4 to 22	51 ± 4/–	CFU/mL: PLPN-CV-15 > PLPN-CV-7.5 > PLPN-15 > PLPN-7.5 > CV-15 > CV-7.5	(65)
Hyaluronic acid-modified micelles with azithromycin and quercetin.	Polymeric	Azithromycin and Quercetin	*Staphylococcus aureus*	RAW264.7 Macrophages	Balb/c mice	74.13 ± 1.25 to 124.47 ± 3.64	–2.63 ± 0.21 to −1.07 ± 0.12	89.55 ± 2.57/2.59 ± 0.13 (AZ) and 2.30 ± 0.15 (Q)	CFU/well: HA-AZI/Qe-M > AZI/Qe-M > AZI-M > AZI	(66)
PLGA (Poly(lactic-*co*-glycolic acid)) nanoparticles	Polymeric	Ciprofloxacin and Ceftazidime	*Klebsiella pneumoniae and Staphylococcus aureus*	RAW264.7 Macrophages and THP-11 Macrophages	–	276 ± 19 to 708 ± 58	–8.41 ± 0.648 to −1.52 ± 0.395	37.8 ± 5.58 to 43.8 ± 4.83/32.9 ± 4.40 to 73.8 ± 13.4	Log (CFU/mL): Cipro L 0.5 > Cipro M 0.5 > Cipro Free > Genta 30 > Genta 100	(67)
Gallium-based metal–organic framework (GaMOF)	Hybrid	Gentamicin	*Staphylococcus aureus*	RAW264.7 Macrophages	Balb/c mice	100	Not specified	Not specified	CFUs (normalized by control): Gen@GaMOFs > GaMOF > Gen	(68)
CA-Dex (Cinnamaldehyde-Dextran) nanoparticles	Polymeric	Glabridin	*Staphyloccocus aureus*	RAW 264.7 Macrophages	Kunming mice	120 ± 10	15.8 ± 0.9	66.1/4.7	Bacterial viability (%). GLA@CA-DEX NPs more effective compared to CA + GLA and GLA	(49)
Cip-CBT-Ada/CD-M (Ciprofloxacin conjugated with cyclodextrin-heptamannoside)	Hybrid	Ciprofloxacin	*Staphyloccocus aureus*	RAW 264.7 Macrophages	Subcutaneous mice infection model	20 ± 2.5	Not specified	Not specified	Bacterial viability (%). CIP (25%), Cip-CBT-Ada (11.7%), Cip-CBT-Ada/CD-M (0.6%)	(69)
Mesoporous Organosilica Nanoparticles (MON) and Mesoporous Silica Nanoparticles (MSN)	Hybrid	Rifampicin	*Staphyloccocus aureus*	RAW 264.7 Macrophages	–	106 ± 12 to 115 ± 6	Not specified	Not specified	Bacterial count (CFU/mL). MON-Rif and MSN-Rif more effective compared to Rif	(70)
Liquid crystalline lipid nanoparticles (LCNP) with dimethyldioctadecylammonium bromide (DDAB)	Lipidic	Tobramycin, Vancomycin	*Staphylococcus aureus, Pseudomonas aeruginosa*	RAW 264.7 Macrophages, A549 lung cells	–	178.7 ± 6.3 to 209.0 ± 2.7	7.73 ± 1.52 to 13.03 ± 0.57	–/4.28 ± 0.84 to 4.91 ± 0.01	CFU/mL and Bacterial viability (%). For P. aeruginosa: LCNP-DDAB and LCNP more effective than Tobramycin in both cell lines; For S. aureus: LCNP-DDAB and LCNP more effective than Vancomysin only in RAW 264.7 cells	(71)
Bacteria-mimetic mesoporous silica nanoparticles (MSN) modified with supramolecular precursors (adamantane and cucurbit[7]uril) and coated with outer membrane vesicles of nonpathogenic *E. coli*	Hybrid	Vancomycin	*Staphylococcus aureus*	RAR 264.7 Macrophages	Mice intraperitoneal infection model	95 to 122	Not specified	20.5	CFU/mL: Mix (SiVPAM + SiVPCM) > SiVPAM ≈ SiVPCM > SiVP > Free Vancomycin	(72)
Monoolein-based cationic cubosomes functionalized with DOTAP (1,2-dioleoyl-3-trimethylammonium-propane)	Lipidic	Rifampicin	*Mycobacterium tuberculosis (MTB-H37Ra)*	THP-1 Macrophages	–	206 to 322	–20 ± 1 to 24 ± 1	78/–	LogCFU/mL: MO–DOTAP-RIF > Free Rifampicin (RIF) > MO–DOTAP > MO	(73)
Poly(a-*N*-acryloyl-phenylalanine)-*block*-poly(b-*N*-acryloyl-d-aminoalanine-*co*-2-*O*-acetyl-a-d-mannosyloxy) nanoparticles	Polymeric	Vancomycin and Curcumin	*Staphylococcus aureus*	RAW 264.7 Macrophages	Balb/c mice peritonitis model	357	–41.4	13.5 to 23.2	Log10CFU: (Van + Cur)@F(AM) NPs > Van@F(AM) NPs > Cur@F(AM) NPs > Free Vancomycin ≈ Free Curcumin	(74)
Neutrophil membrane-coated PLGA nanoparticles (KLA-NNPs) loaded with gentamicin and functionalized with the KLA peptide	Hybrid	Gentamicin and KLA peptide	*Klebsiella pneumoniae*	MLE12 mouse lung epithelial cell	C57BL/6 mice	95.69 ± 3.81 to 311.83 ± 5.11 nm	–31.4 ± 1.08 to −17.4 ± 1.23	Not specified	CFU/well: KLA-NNPs > KLA > NNPs > Gentamicine	(34)
Fucoidan-coated nanoparticles (FU/ML-LA/EB NPs) composed of metformin-linoleic acid (ML), linoleic acid (LA), and ebselen (EB)	Hybrid	Ebselen	*Helicobacter pylori*	RAW 264.7 Macrophages and GES-1 gastric cells	C57BL/6 mice gastric infection model	127.1 ± 2.2 to 152.6 ± 1.4	–51.8 ± 1.9 to +40.8 ± 0.4	81.88 ± 0.31 to 85.44 ± 0.40/–	Bacterial viability (%). FU/ML-LA/EB NPs > ML-LA/EB NPs > ML + LA + EB > ML > MET	(75)
Mannose-decorated poly(α-*N*-acryloyl-phenylalanine)-*block*-poly(β-*N*-acryloyl-d-aminoalanine) nanoparticles loaded with rifampicin.	Polymeric	Rifampicin	*Staphylococcus aureus*	THP-1 Macrophages	Balb/c mice peritonitis model	250 to 280	–30.6 to −29.4	–/18.9	Log10 CFU: Rif@FAM NPs > Rif@FM NPs > Rif@FM NPs > Rif	(76)
Tetraphenylethylene-poly(2-methacrylamido mannopyranose-random-2-lactobionamidoethyl methacrylate)-*block*-poly(ε-caprolactone)-*block*-poly(3-acrylamidophenylboronic acid) (TPE-PMAMA-r-PLAMA/PCL-*b*-PAAPBA) (T-r/40)	Polymeric	Clarithromycin	*Staphylococcus aureus*	RAW 264.7 Macrophages	Balb/c mice	150 to 190	–20	–/10.2	Intracellular CFUs: T-r/40@CLA > CLA > T-r/40	(77)
Phosphatidylserine (PS)-coated poly[(4-allylcarbamoyl-phenylboric acid)-*ran*-(arginine-methacrylamide)-*ran*-(*N*,*N*′-bisacryloylcystamine)] nanoparticles (PABS).	Hybrid	Vancomycin	*Staphylococcus aureus*	RAW 264.7 Macrophages	Mouse model	40	–9 to −6	–/11.0	Bacterial viability (%)	(78)
SIR-micelles(+) composed of F127 copolymer with modifications for increased hydrophobicity	Polymeric	Colistin	*Klebsiella pneumoniae*	J774A.1 Macrophages	CD-1 mice	190	10	38–40/–	CFU/mL (Log10): SIR-micelles(+) > SIR-micelles > SIS-micelles > NPC-micelles > Free colistin	(79)
Poly(ethylene oxide)-polycaprolactone (PEO–PCL) block copolymers forming polymersomes encapsulating doxycycline or rifampicin	Polymeric	Doxycycline and rifampicin	*Burkholderia thailandensis*	RAW 264.7 Macrophages	–	100 to 150	Not specified	Not specified	CFU/mL: PM-doxy > doxy > PM-empty; PM-rif > rif > PM-empty	(80)
ROS-sensitive micelles containing amphiphilic diblock copolymers of poly(ethylene glycol) (PEG) and phenylboronic pinacol ester blocks, encapsulating rifampin (RIF)	Polymeric	Rifampincin	*Staphylococcus aureus*	J774A.1 Macrophages	–	50 to 58	Not specified	52–53/6.1–6.2	CFU/mL: MF/RIF > RIF > mPEG-*b*-PS/RIF	(81)
Mannosylated glycoparticles containing pH-responsive isoniazid monomers	Polymeric	Isoniazid	*Mycobacterium bovis BCG*	THP-1 Macrophages	–	131 to 280	Not specified	–/33–48	CFU: Mannose-hydrazone-INH > Isoniazid (INH) > PEGA-hydrazone-INH > Mannose-urea-INH = Mannose no INH	(82)
Dendritic mesoporous silica nanoparticles (DMSN)	Inorganic	NapFab (an optimized derivative of a Napsin A fragment)	*Mycobacterium tuberculosis*	Primary human macrophages	Zebrafish embryos (toxicity experiments)	163 ± 7	–17.5 ± 0.4	–/27 ± 1 to 28 ± 1	Log10(normalized growth): NapFab@DMSN > NapFab > DMSN > MSFM	(83)
Poly(lactic-*co*-glycolic acid) (PLGA) particles decorated with silver (Ag) nanoparticles and loaded with the antimicrobial peptide pexiganan	Hybdrid	Pexiganan	*Staphylococcus aureus, Listeria monocytogenes, Salmonella typhimurium and Shigella flexneri*	J774A.1 Macrophages	Balb/c mice	603.2 ± 37.3	–23.5 ± 1.2	5/–	Log (CFU/mL): Pex@NP-pTA-Ag > NP-pTA-Ag > Pex@NP > PEX	(84)
Lactoferrin nanoparticles	Protein-based	Berberine, Sanguinarine, Vancomycin, Imipenem	*Staphylococcus aureus, Mycobacterium abscessus*	THP-1 Macrophages	–	191.8 ± 11.1	8.03 ± 3.84	68.3 ± 1.84 to 87.9 ± 2.56	Log10 (CFU/mL) – 50 μg/mL	(85)
Polycationic micelles formed by mPEG-*b*-PP copolymer with PEI layers, encapsulating tetracycline	Polymeric	Tetracycline	*Escherichia coli (EB1–1)*	RAW 264.7 Macrophages	Balb/c mice	99.4 ± 7.1 to 102.5 ± 8.6	–12.7 ± 0.87 to +18.2 ± 0.31	90.7 ± 4.3/19.5 ± 2.1	% killing of intracellular bacteria: PP–PEI/TC > mPP/TC > TC	(86)
Mesoporous silica nanoparticles (MSN)	Inorganic	Rifampicin	*Staphylococcus aureus*	Caco-2 colon cells	–	47.0 ± 7.0 to 84.1 ± 17.4	–15.1 ± 5.4 to −13.6 ± 6.3	–/28.9 to 38.2	CFU/mL: Rif-MSN40e > Rif-MSN80c > Rif-MSN40c > Rif	(87)
Gentamicin-decorated phosphatidylcholine-chitosan nanoparticles (GPC NPs)	Polymeric	Gentamicin	*Listeria monocytogenes and Pseudomonas aeruginosa*	RAW264.7 Macrophages	–	∼137.3	–19.5	Not specified	Bacteria count (CFU × 10^6^): GPC NPs > GEN > SIM	(46)
Mannose-grafted polymer nanoparticles encapsulating a siderophore-antibiotic conjugate (DFO–CIP) and Fe^3+^ ions	Polymeric	Ciprofloxacin and deferoxamine	*Staphylococcus aureus*	RAW 264.7 Macrophages	C57Bl/6 mice	105 to 141	Not specified	–/10.7 to 42.7	CFU/well: mPET@DFeC > mPET@DFeC/Man > PET@DFeC > DFeC > mPET	(88)
Poly(lactic-*co*-glycolic acid)-lipid hybrid microparticles (PLH) loaded with rifampicin	Hybrid	Rifampicin	*Staphylococcus aureus*	RAW 264.7 Macrophages	–	110 ± 11 to 5390 ± 110	–15.3 ± 1.2 to −7.12 ± 1.5	24.2 ± 0.1 to 47.4 ± 4.2/1.21 ± 0.1 to 11.7 ± 0.4	CFU/mL: Rif-PLH > Rif-PLGA > Rif	(89)
Beta-cyclodextrin (BCD) combined with oleylamine (OLA) to form supramolecular amphiphilic nanovesicles, encapsulating vancomycin	Hybrid	Vancomycin	*Staphylococcus aureus*	THP-1 Macrophages and HEK 293 kidney cells	–	104.6 ± 0.68 to 210.9 ± 50.00	19.3 ± 9.20 to 45 ± 0.95	31.0 ± 1.00 to 51.6 ± 2.30/7.3 ± 2.30 to 15.5 ± 1.00	Log10 CFU/mL: BCD-OLA 5× MIC > BCD-OLA MIC > VCM 5× MIC > VCM MIC for both cell lines	(90)
Penicillin G-derived phospholipid nanoparticles (PenG-PL NPs) composed of Penicillin G conjugated with phospholipids, PEG, and cholesterol	Hybrid	Penicillin G and Levofloxacin	*Staphylococcus aureus*	A549 lung cells	–	Not specified	Not specified	48.2 ± 3.9	%Loss of log10CFU: PenG-PL NPs > PenG	(91)
Chitosan nanoparticles	Polymeric	Gentamicin	*Brucella melitensis and Brucella abortus*	J774A.1 Macrophages	–	70 to 100	28	–/22 to 72	Mean CFU: Gen-Cs > Free Gen > Bare NPs	(92)
Lipid–dendrimer hybrid nanoparticles (LDH-NPs) composed of oleylamine (OLA), poly(amidoamine) dendrimer (PAMAM G3), and vancomycin (VCM)	Hybrid	Vancomycin	*Staphylococcus aureus*	HEK 293 kidney cells	–	124.4 ± 2.01 to 252.7 ± 3.98	–7.15 ± 2.98	82.70 ± 4.09/–	MRSA Log10CFU/mL: LH-DNPs > Bare VCM	(93)
Liposomes composed of DPPC, DSPC, DPPE-GA, and cholesterol, encapsulating colistin and functionalized with extracellular adherence protein (Eap)	Lipidic	Colistin	*Salmonella enterica*	Hep-2 epithelial and Caco-2 colon cells	–	201.3 ± 1.0 to 211.8 ± 1.7	–21.0 ± 0.6 to – 15.3 ± 1.2	55.3 ± 5.2 to 61.7 ± 5.7/49.8 ± 0.4 to 50.9 ± 0.7	Bacterial killing (%): EapCol-Lip-3 > Col-Lip-3 > Lip-3 > Colistin	(94)
Glucosamine/l-lactide (GluN-LLA) copolymers loaded with rifampicin (RIF)	Polymeric	Rifampicin	*Mycobacterium smegmatis*	J774A.1 Macrophages	–	500 ± 30 to 2100 ± 600	Not specified	15.0 ± 0.2 to 71.0 ± 1.7/-	CFU/cell: MP-GluN-LLA > MP-LLA > SMP-GluN-LLA > SMP-LLA > RIF	(95)
Mesoporous silica nanoparticles (MSNPs) synthesized as Hiroshima mesoporous material (HMM), encapsulating rifampicin	Inorganic	Rifampicin	*Staphylococcus aureus*	RAW 264.7 Macrophages	–	47.5 ± 4.8 to 78.4 ± 5.7	–20.0 ± 7.36 to −16.9 ± 3.45	22.5 to 26.8/38.3 to 41.1	CFU/mL: MSNP-Rif 100 > MSNP-Rif 40 > Rif	(96)
Polyhexamethylene biguanide (PHMB) combined with nadifloxacin, forming self-assembled nanoparticles	Polymeric	Nadifloxacin	*Staphylococcus aureus*	HaCaT keratinocytes cell	–	291.3 ± 89.6	+20.2 ± 4.83	58/–	% survival rate: PHMB > PHMB/nadifloxacin NPs > PHMB + nadifloxaci*n* = nadifloxacin = PHMB > nadifloxacin	(97)
Hyaluronan-streptomycin conjugated with decylamine and encapsulating rapamycin	Polymeric	Streptomycin	*Salmonella typhimurium and Staphylococcus aureus*	RAW 264.7 Macrophages	–	130 to 179	–9.8 to −18.9	69.3/–	Log CFU: HAASD-Rapa > Strep+Rapa > Strep > Rapa	(98)
Beta-glucan particles (GP) encapsulating nanorifabutin (RB)	Polymeric	Rifabutin	*Mycobacterium tuberculosis*	J774A.1 Macrophages	–	–	–	40.5/–	Log CFU/mL surviving: RB-NPs-GP > Soluble RB > Blank GP	(99)
Disordered mesoporous silica particles (MSPs)	Inorganic	Clofazimine	*Mycobacterium tuberculosis*	THP-1 Macrophages, Calu-3 lung cells	–	2,430 with pore size 9–10	Not specified	3 to 10	MIC99: CLZ (10 μg/mL), CLZ-MSPs (1.25 μg/mL)	(100)
Polyanhydride nanoparticles synthesized from copolymers of sebacic acid, 1,6-bis(*p*-carboxyphenoxy)hexane (CPH), and 1,8-bis(*p*-carboxyphenoxy)-3,6-dioxaoctane (CPTEG)	Polymeric	Doxycycline and rifampicin	*Brucella melitensis*	RAW264.7 Macrophages	Balb/c mice	162.8 ± 52.0 to 326.8 ± 117.8	–21.2 ± 1.8 to −1.56 ± 7.6	19.6 ± 0.7 to 100/–	Log 10 CFU/mL: CPTEG:CPH > CPH:SA > Soluble	(101)
Poly(lactic-*co*-glycolic acid) (PLGA) nanoparticles functionalized with InvA497 (a fragment of the Yersinia invasin protein) and loaded with AOT-gentamicin (a lipophilic form of gentamicin)	Polymeric	AOT-gentamicin	*Shigella flexneri*	Hep-2 epithelial cells	–	164.8 ± 8.3	–16.5 to −14.8	33 to 43/–	Bacterial killing (%): AsphIG > SphIG > G > AsphI = AsphG = Asph = SphI = SphG = Sph	(102)
Poly(lactic-*co*-glycolic acid) (PLGA) nanoparticles encapsulating gentamicin	Polymeric	Gentamicin	*Klebsiella pneumoniae*	MH-S Macrophages and THP-1 cells	Galleria mellonella	227	–1.67	–/13.5	CFU/well: GNPs > BNPs = Free G	(103)
Hyaluronan-cholesterol nanohydrogels (HA-CH NHs) loaded with gentamicin (GM) or levofloxacin (LVF)	Polymeric	Gentamicin and Levofloxacin	*Staphylococcus aureus*	HaCaT keratinocytes cell	–	≈250 to 300	Not specified	7.9 ± 0.8 to 40.0 ± 1.0/2.0 ± 0.2 to 40 ± 1.0	Log10 CFU/mL: NH/LVF > NH/GM > GM > LVF > NHs	(104)
Nanogels (NGs) composed of phenylboronic acid-modified ε-polylysine and tannic acid, loaded with hydroxyapatite nanoparticles (mHA G) and gentamicin (GEN	Hybrid	Gentamicin	*Staphylococcus aureus*	RAW 264.7 Macrophages	Balb/c rats	79 to 106	–27.7 to 9.4	27.8/6.9	Bacterial viability (%): GEN (84.2%), mSNG (66.1), mHAG (20.6%), NG@G1-mHAG2 (1%)	(105)
Gold nanoparticles (AuNPs) conjugated with polymyxin B sulfate (PMB) and sushi peptide, functionalized with folic acid for targeting	Inorganic	Polymyxin B and sushi peptide	*Salmonella typhi*	HeLa cervical cells	–	16 ± 4	Not specified	∼95/–	Vaibility (%): AuNPs-Sh 1:1000 > 1:2000 > 1:700 > 1:500 > Sushi > AuNPs	(106)
Phosphatidylcholine-decorated gold nanoparticles (AuNPs) loaded with gentamicin (GPA NPs)	Hybrid	Gentamicin	*Pseudomonas aeruginosa and Listeria monocytogenes*	RAW264.7 Macrophages	–	180	–34.0 to −24.7	Not specified	Bacterial count (CFU): GPA > GEN > PA	(107)
Liposomes functionalized with InvA497 (a C-terminal fragment of invasin from *Yersinia pseudotuberculosis*), composed of DPPC, cholesterol, and a phosphoethanolamine derivative	Lipidic	Gentamicin	*Yersinia pseudotuberculosis*	Hep-2 epithelial cells	–	140.6 ± 0.4 to 144.6 ± 1.7	–42.9 ± 0.6 to −19.9 ± 1.7	Not specified	Bacterial killing (%): I-GL > I-L > L > GL	(108)
Mesoporous silica nanoparticles (MSNs) functionalized with pH-sensitive nanovalves	Inorganic	Moxifloxacin	*Francisella tularensis*	THP-1 Macrophages	Balb/c mice	100	–46.28 to 38.76	51.4/–	Log CFU per monolayer: MSN-MBI-MXF > Free MXF	(109)
Van-DM NPs (disulfide-vancomycin nanoparticles)	Polymeric	Vancomycin	*Staphylococcus aureus*	RAW 264.7 macrophages, NIH/3T3 fibroblast cells	ICR mice	80	17	36/–	Bacterial viability (%). Van-DM NPs more effective compared to VAN and DM	(110)
Mesoporous silica nanoparticles (MSNs) functionalized with poly(ethylene imine)–poly(ethylene glycol) (PEI–PEG) and loaded with isoniazid (INH)	Hybrid	Isoniazid	*Mycobacterium tuberculosis*	THP-1 Macrophages	Balb/c mice	50 to 100	Not specified	–/8.2 to 11.3	Log CFU per monolayer: MSN–CHO–INH–PEI–PEG > SMSN–CHO–INH–PEI–PEG > Free INH	(111)
Clindamycin-loaded calcium phosphate nanoparticles (Hydroxyapatite (HAP), Amorphous Calcium Phosphate (ACP), Dicalcium Phosphate (DCPA))	Inorganic	Clindamycin	*Staphylococcus aureus*	MC3T3-E1 preosteoblastic cell	–	62 to 84	Not specified	–/1 to 5	Colony count: ACP/CL > HAP/CL > CL > DCPA/CL	(112)

**Figure 2:**
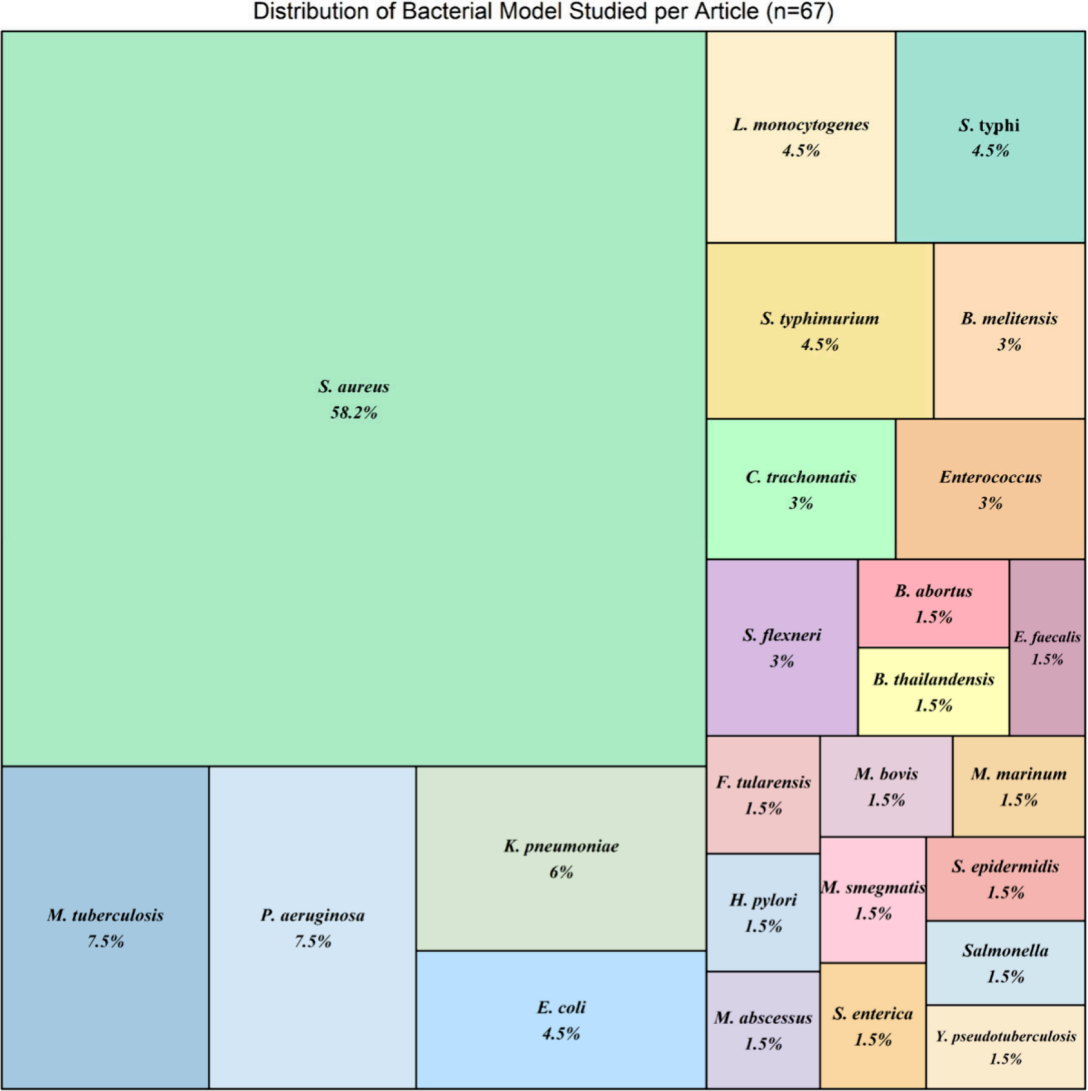
Treemap illustrating the distribution of bacterial models studied across 67 reviewed articles. Each rectangle represents a bacterial species with its size proportional to its frequency of study.

The most commonly studied bacterium was *S. aureus*, which was used in 58.2% of the studies (*n* = 39). Other frequently studied pathogens included *P. aeruginosa* and *M. tuberculosis*, each accounting for 7.5% of the studies (*n* = 5) followed by *Klebsiella pneumoniae* with 6% (*n* = 4). *Salmonella typhi*, *L. monocytogenes*, *E. coli*, and *S. typhimurium* were each examined in 4.5% of the studies (*n* = 3). Additional bacterial models were evaluated in fewer than 3% of the studies (Figure 2).

**Figure 3:**
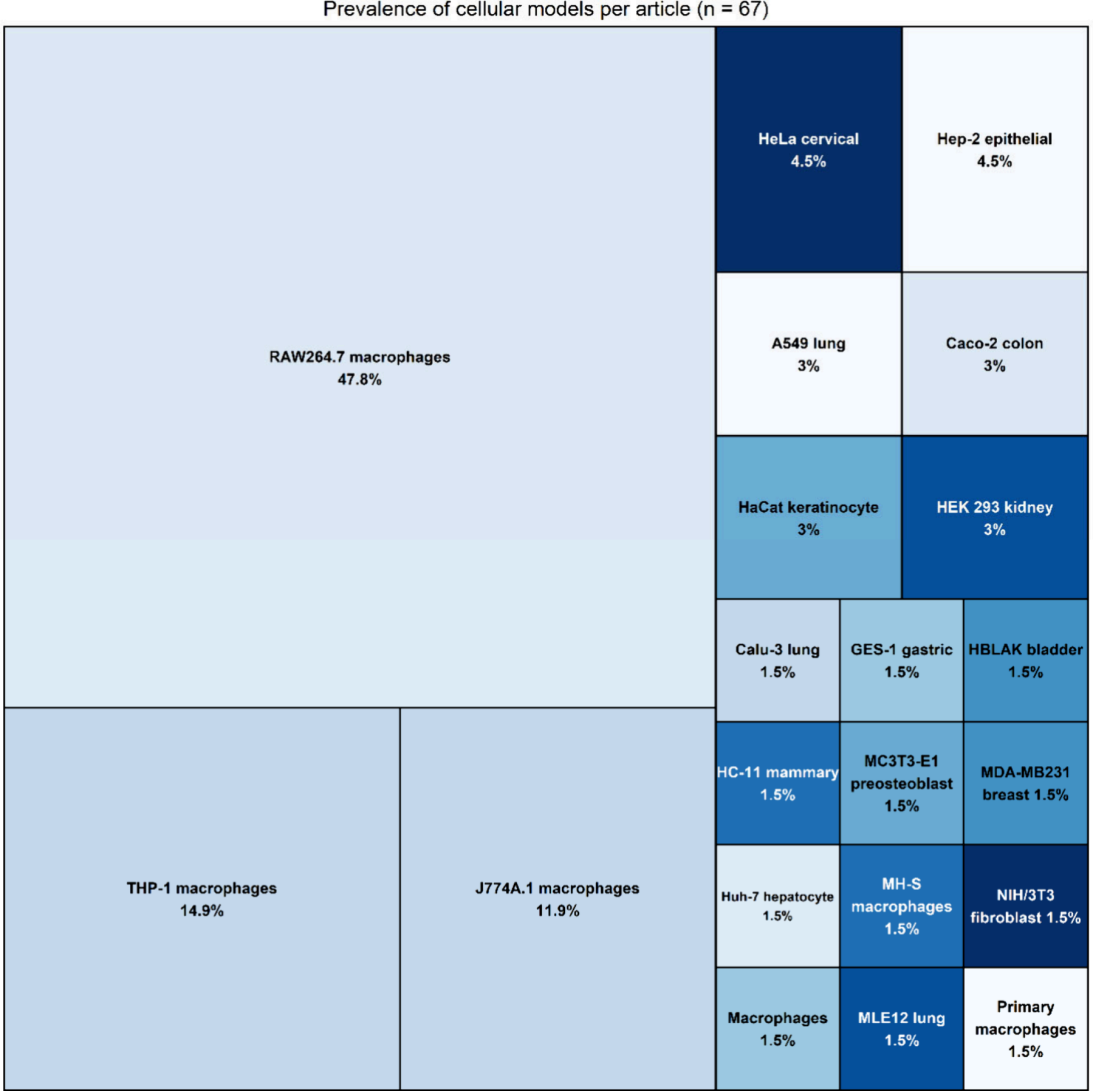
Treemap illustrating the prevalence of cellular models used across 67 reviewed articles. Each rectangle represents a specific cellular model with the size proportional to its frequency of use.

For intracellular evaluations, macrophages were the primary cellular models, with RAW264.7 cells being the most utilized (47.8%, *n* = 32), followed by THP-1 (14.9%, *n* = 10) and J774A.1 (11.9%, *n* = 8) cell lines. Other cellular models were utilized in less than 5% of the studies (Figure 3).

*In vivo* evaluations of the nanoparticle system efficacy were conducted in 30 studies (44.8%), employing various animal and infection models (Table 1).

**Figure 4:**
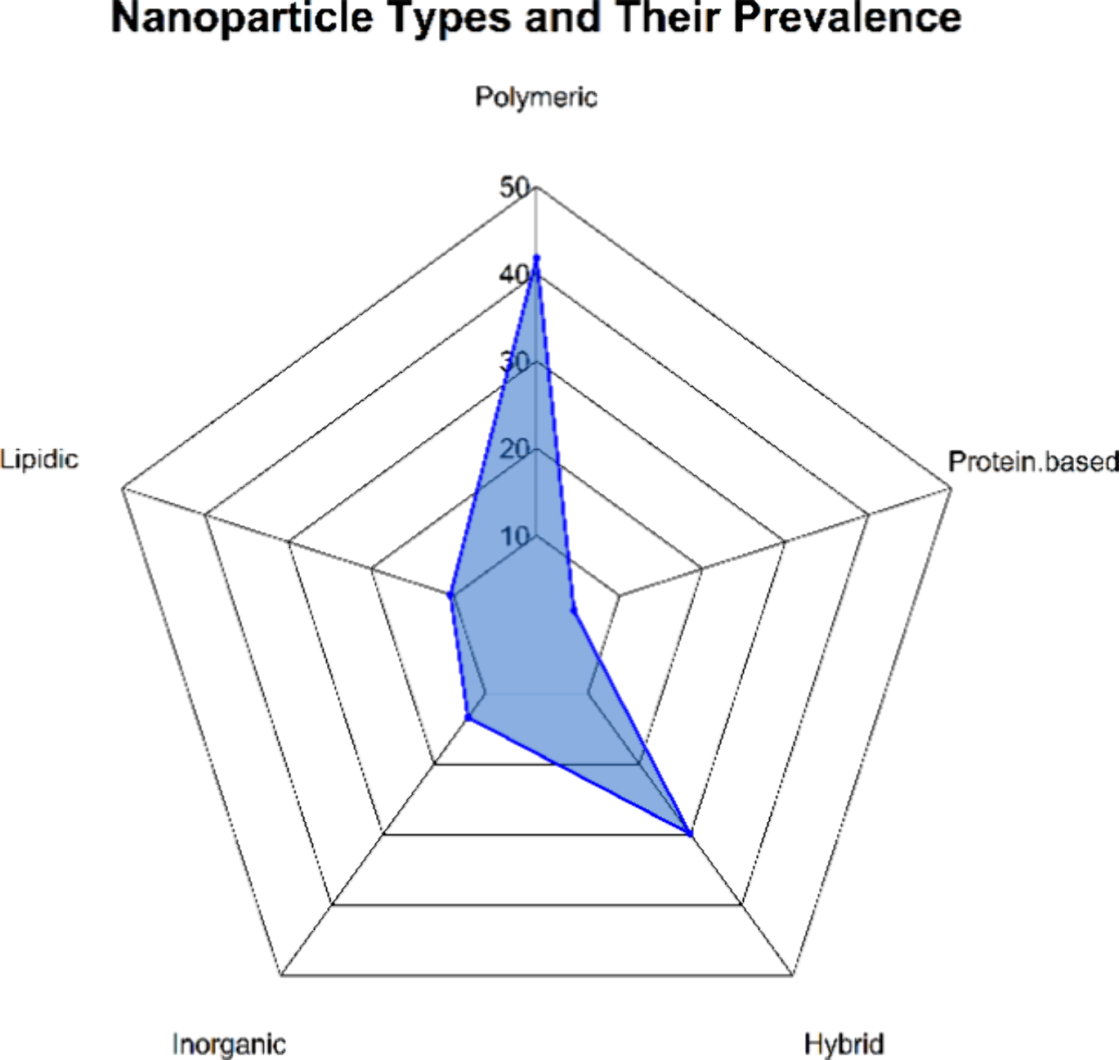
Spider plot showing the prevalence of different types of nanoparticles reported in 67 reviewed articles. The axes represent the percentage of prevalence for each type of nanoparticle type.

A wide variety of nanoparticles were used for the delivery of antibacterial agents, mainly polymeric nanoparticles (41.8%), followed by hybrid nanoparticles, a combination of different types of materials (29.9%), inorganic (13.4%), lipidic (10.4%), and protein-based (4.5%) nanoparticles (Figure 4).

**Figure 5:**
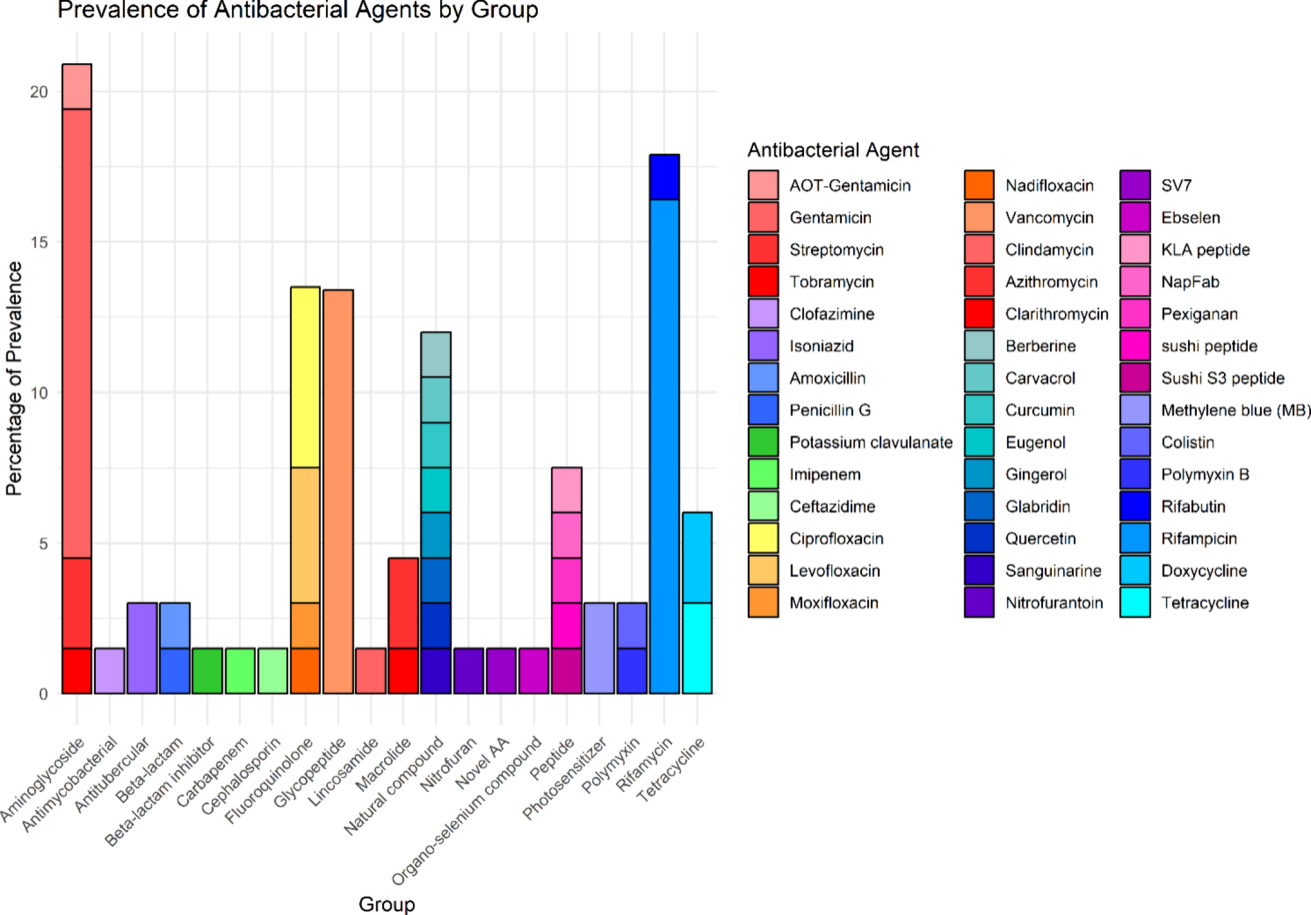
Stacked bar chart representing the prevalence of antibacterial agents grouped by their chemical or functional class across 67 reviewed articles. Each bar segment corresponds to a specific antibacterial agent with the height representing its percentage of prevalence within the group. Aminoglycoside: AOT-Gentamicin, Gentamicin, Streptomycin, and Tobramycin; Antimycobacterial: Clofazimine; Antitubercular: Isoniazid; Beta-lactam: Amoxicillin, Penicillin G; Beta-lactam inhibitor: Potassium clavulanate; Carbapenem: Imipenem; Cephalosporin: Ceftazidime; Fluoroquinolone: Ciprofloxacin, Levofloxacin, Moxifloxacin, Nadifloxacin; Glycopeptide: Vancomycin; Lincosamide: Clindamycin; Macrolide: Azithromycin, Clarithromycin; Natural compound: Berberine, Carvacrol, Curcumin, Eugenol, Gingerol, Glabridin, Quercetin, Sanguinarine; Nitrofuran: Nitrofurantoin; Novel AA: SV7; Organo-selenium compound: Ebselen; Peptide: KLA peptide, NapFab, Pexiganan, sushi peptide, and sushi S3 peptide; Photosensitizer: Methylene blue; Polymyxin: Colistin, Polymyxin B; Rifamycin: Rifabutin, Rifampicin; Tetracycline: Doxycyline, Tetracycline.

These nanoparticulate systems were primarily used for the delivery of conventional antibiotics, natural compounds like phenols and antibacterial peptides, with aminoglycosides being the most studied (20.9%), followed by rifamycins (17.9%), fluoroquinolones (13.4%), and glycopeptides (13.4%), natural compounds (11.9%), antimicrobial peptides (7.5%), tetracyclines (6.0%), macrolides and polymyxins (4.5%), antitubercular agents and beta-lactams (3.0%), organo-selenium compounds, antimycobacterials, cephalosporins, beta-lactamase inhibitors, photosensitizers, nitrofurans, lincosamides, and novel antibacterial agents (1.5%). Specifically, gentamicin, vancomycin, and rifampicin were the most utilized (Figure 5). Although all of the reviewed articles report an improvement in the efficacy of antibacterial agents and nanoparticles against intracellular bacteria, the way results are presented is highly variable, primarily in the form of CFU/mL and viability percentages. This variability complicates comparisons between studies, as most articles fail to report an MIC (minimum inhibitory concentration) or MBC (minimum bactericidal concentration) value. Notably, most articles do not report complete eradication of intracellular bacteria, a factor that could contribute to recurrent infections. However, such recurrences were not assessed due to the limited time frame of the studies.

## Discussion

Intracellular bacterial infections pose a significant therapeutic challenge due to the physical barriers created by host cell membranes and intracellular compartments, which hinder effective antibiotic delivery. While nanotechnology and nanoparticle-based systems have been extensively studied, their potential to enhance the efficacy of antibacterial agents specifically targeting intracellular pathogens remains underexplored. This systematic review highlights the promising role of nanoparticle-based systems in addressing these challenges and provides a foundation for future research and clinical applications.

Understanding the role of intracellular bacterial infections requires examining the pathogens most frequently associated with these infections and their unique challenges, as highlighted in the reviewed studies.

*Staphylococcus aureus* emerged as the most frequently studied pathogen, reflecting its clinical relevance and the urgent need for innovative therapies to combat its multidrug-resistant strains. *S. aureus* is responsible for pathologies, such as osteomyelitis, endocarditis, and skin infections, potentially leading to bacteremia and sepsis. The World Health Organization (WHO) has reported methicillin-resistant *S. aureus* (MRSA) incidences ranging from 20% to 80%, with high associated mortality rates.^17^ Additionally, *S. aureus* can form persistent phenotypes such as small colony variants (SCVs), characterized by low metabolic activity and resistance to antibiotics. SCVs can invade both professional phagocytic cells, like macrophages, and nonprofessional phagocytic cells, such as epithelial cells, providing a mechanism for persistence and evasion of the immune system.^18,19^

Despite *S. aureus* being one of the priority pathogens listed in the WHO 2024 priority pathogens list,^20^ other high-priority pathogens such as *P. aeruginosa*, *K. pneumoniae*, and *E. coli* also possess the ability to invade host cells. However, their representation in the reviewed studies was notably low, indicating a gap in the research. Similarly, *Acinetobacter baumannii*, a critical pathogen in healthcare-associated infections due to its antibiotic resistance and ability to invade and persist within cells, was absent in the studies, representing a significant opportunity for scientific exploration^21^*Mycobacterium tuberculosis*, one of the most clinically relevant intracellular pathogens, was featured in only 5 of the 67 reviewed studies, while other *Mycobacterium* species appeared in just 4 studies. Obligate intracellular bacteria, such as *C. trachomatis* was only studied in 2 articles, while *Rickettsia* spp. was not studied underscoring the need for broader pathogen coverage in future research.^22^

Given the complexity of these pathogens and their interactions with host cells, selecting appropriate experimental models becomes critical to uncovering mechanisms of infection and evaluating therapeutic interventions.

The use of cell lines in infectious disease research provides a controlled environment to study microorganism-cell interactions, the mechanisms of infectious processes, and microorganism-cell-antibiotic interactions. Therefore, selecting appropriate cell lines that closely mimic bacterial behavior in a complete organism is crucial. The most frequently used cell lines were macrophage-derived, predominantly murine, such as RAW264.7 and J774A.1 (59.7%). However, microorganism interactions may differ between murine and human models,^23^ which could be significant, especially considering that nanoparticle-cell line interactions might also vary. The studies reviewed displayed substantial variability in the cell lines used, which aligned with the diversity of bacterial species studied. Each species can infect and colonize different tissues and cell types, but this variability limits the comparability of results and, consequently, the assessment of nanoparticle system efficacy under specific experimental conditions and between studies, even when the same antibacterial agent was used.

Despite their advantages, *in vitro* models cannot replicate the systemic dynamics of infections; *in vivo* models are indispensable for understanding host responses, pharmacokinetics, and biodistribution of nanoparticle-based systems. Only 44.8% of the reviewed articles employed *in vivo* models, emphasizing the need to increase this percentage to capture the complexity of infectious processes. For instance, while macrophage cell lines like RAW264.7 offer valuable information on intracellular bacterial persistence, *in vivo* models enable the evaluation of immune cell recruitment, tissue-specific responses, and systemic factors like cytokines or blood flow, which are absent in cell cultures.^24^

Furthermore, *in vivo* models are indispensable for assessing nanoparticle-based systems’ biodistribution, biocompatibility, and therapeutic efficacy.^25,26^ Studies using mouse models have demonstrated how nanoparticles can effectively target infected tissues, offering insights into their potential for site-specific drug delivery. However, the small number of *in vivo* studies is an opportunity that requires immediate attention.

One of the key challenges in advancing nanoparticle technologies for intracellular infections is the lack of standardization in experimental methodologies, particularly in evaluating the nanoparticle efficacy across diverse cellular and animal models. This variability in approaches can lead to inconsistent results, making it difficult to compare studies and assess the true potential of nanoparticle-based therapies.

The most commonly used method for evaluating the activity of compounds against intracellular bacteria *in vitro* is the invasion assay, also known as the gentamicin protection assay.^27^ In this technique, host cells are infected with the bacterial strain of interest, allowing time for internalization, after which noninternalized bacteria are eliminated using high concentrations of antibiotics, typically gentamicin. In this context, Subramaniam et al. (2024) recently published a review highlighting the minimum essential information that should be reported for intracellular infection assays. These include the cell line used, bacterial strain, multiplicity of infection (MOI), infection time, antibiotic used, its concentration and exposure duration, as well as the treatment time with the agents under evaluation.^28^ Additional details, such as the type of culture medium used, number of washing steps, centrifugation time and speed, and types of controls, can also be reported to enhance reproducibility. In the case of the studies reviewed here, this information was extracted from the experimental sections. However, it was found that not all articles provide complete methodological details (Table S1), and in some cases, critical information such as the MOI, infection time, or even number of seeded cells is missing. This absence of methodological detail hinders the reproducibility of experiments and, in particular, complicates the comparison of the results across different studies. Addressing these limitations through standardized protocols and increased *in vivo* research is critical for clinical translation. Standardization allows for more accurate comparisons between studies, facilitating a deeper understanding of how nanoparticles behave in biological environments. The insights gained from these models provide a strong foundation for designing nanoparticle systems tailored to overcoming the unique challenges posed by intracellular infections.

Nanoparticle systems offer significant advantages in the treatment of intracellular infections. By protecting encapsulated agents and enabling targeted delivery, these systems address challenges posed by physical and biological barriers. For instance, *M. tuberculosis* typically resides within pulmonary cells, requiring nanoparticles capable of accumulating in the lungs and interacting with pulmonary tissues. Similarly, *E. coli*, a common cause of urinary tract infections, can invade bladder tissues and internalize within epithelial cells to form intracellular bacterial communities (IBCs). Therefore, nanoparticles designed to treat such infections must reach the urinary tract and accumulate in sufficient concentrations within the bladder.

Since the treatment of each microorganism has specific requirements, the design of nanoparticle systems is a crucial factor in selecting the appropriate therapeutic platform. Polymeric nanoparticles dominated the studies reviewed, accounting for 41.8% of the nanoparticle systems used. Their popularity arises from their versatility in encapsulating a wide variety of antibacterial agents and their ability to achieve localized delivery through higher drug accumulation at target sites.^29^ However, polymeric nanoparticles are associated with cytotoxicity concerns and challenges in large-scale production.

In contrast, lipid nanoparticles, comprising 10.4% of the systems studied, offer lower toxicity due to their natural, biocompatible components. These systems are particularly effective in improving drug permeation across biological barriers, making them suitable for applications requiring systemic delivery.^30^ However, they are underrepresented in research, partly due to stability limitations and a lack of commercially available formulations.

The choice between polymeric and lipid nanoparticles should ultimately depend on the infectious process of interest. Polymeric nanoparticles may be more advantageous for localized infections requiring sustained drug accumulation, such as skin or mucosal infections, while lipid nanoparticles are better suited for systemic delivery to address intracellular infections requiring efficient penetration across barriers.^31,32^ Future research should focus on optimizing these systems to tailor them to the specific requirements of different bacterial pathogens and infection sites.

Additionally, hybrid nanosystems that combine features from various molecular types have been shown to improve drug delivery. For example, metal–organic frameworks (MOFs) enable efficient drug encapsulation while contributing antibacterial activity through the release of metal ions. When combined with polysaccharides such as hyaluronic acid, they gain specificity for CD44 receptors on the surface of macrophages.^33^ Another study implemented neutrophil membrane-coated PLGA nanoparticles, which offer the advantages of polymeric nanoparticles but, by being coated with cell membranes, mimic natural cells, enabling them to evade recognition and clearance by the immune system.^34^

Despite the promise of nanoparticle-based approaches, understanding the limitations of conventional antibiotics highlights the need for innovative delivery systems to enhance their efficacy.

Beyond the choice of nanoparticle type, their physicochemical properties play a fundamental role in determining their effectiveness against intracellular infections. Cellular uptake mechanisms are highly dependent on particle size as this parameter influences the type of endocytic pathway engaged during internalization. Particle size therefore exerts a significant impact on both the route of cellular entry and subsequent intracellular trafficking—factors that are critical for the therapeutic success of drug delivery systems.^35^ It is well established that smaller nanoparticles (<200 nm) are predominantly internalized via clathrin- and caveolae-mediated endocytosis, directing them primarily toward endosomal and lysosomal compartments. This lysosomal targeting is advantageous in infections where pathogens reside within these organelles, as it promotes localized drug release and enhanced therapeutic efficacy.^36,37^ In contrast, larger particles (>200 nm), particularly those around 1 μm in diameter, are more likely to be internalized through caveolin-mediated endocytosis, macropinocytosis, or phagocytosis—pathways that may allow particles to evade lysosomal degradation and favor cytosolic delivery. Such mechanisms are especially relevant for pathogens that exploit nonlysosomal intracellular niches. Several studies, including those by Maghrebi et al. (2023) have demonstrated that microparticle-based delivery systems can be highly effective in this context, with particles around 1 μm offering optimal uptake by phagocytic cells and improved intracellular distribution.^38^

Additionally, the surface charge—or zeta potential—of the particles plays a critical role in cellular interaction and internalization. Positively charged nanoparticles tend to interact more strongly with the negatively charged cell membranes, promoting uptake. However, excessive positive charge (and even negative charges) can lead to opsonization and rapid clearance due to protein corona formation, which in turn can modify cellular interactions and affect the targeting ability of the system. Thus, a delicate balance between size and surface properties is necessary to achieve effective intracellular delivery.^36,39^ Despite the critical importance of determining the surface charge (zeta potential) of nanoparticle systems—given its influence on cellular interaction, stability in biological media, and internalization efficiency—not all studies included in this review report this parameter.

Beyond passive uptake mechanisms, active targeting strategies based on ligand–receptor interactions offer an additional layer of specificity and can significantly enhance the delivery of antimicrobials to infected cells. These strategies are particularly valuable when dealing with complex tissue environments or heterogeneous cell populations, as they enable selective uptake and may direct the particles toward endocytic routes that favor therapeutic outcomes.^40^

Intracellular bacterial infections pose a significant therapeutic challenge due to the physical barriers created by host cell membranes and intracellular compartments, which hinder effective antibiotic delivery.^41^ Many commonly used antibiotics, such as β-lactams and aminoglycosides, exhibit poor intracellular penetration or retention. β-lactams struggle to diffuse across lipid bilayers due to their polar nature, while aminoglycosides are often sequestered in endosomes or expelled via exocytosis, limiting their efficacy.^5,42^ Conversely, certain antibiotic classes, including macrolides, fluoroquinolones, tetracyclines, and rifamycins, demonstrate superior intracellular activity.^43,44^ These antibiotics can penetrate host cells via passive diffusion and reach effective concentrations within subcellular compartments, such as acidified phagosomes, where intracellular pathogens reside. Notably, fluoroquinolones and rifamycins are particularly effective due to their ability to accumulate in macrophages and target bacteria within cytosolic or vacuolar niches.^45^ Emerging strategies, such as nanoparticle-based drug delivery, offer promising solutions by enhancing intracellular transport and the targeted release of antibiotics, further addressing the limitations of conventional therapies and providing hope for more effective treatment of intracellular infections.

All of the studies presented in this review enhanced the antibacterial efficacy of different antibiotics against intracellular bacteria upon encapsulation or functionalization with a variety of nanoparticle systems. For instance, it is well-known that gentamicin, an aminoglycoside, is incapable of penetrating the interior of eukaryotic cells. This characteristic makes it a common tool in gentamicin protection assays, where it effectively eliminates extracellular bacteria without affecting intracellular pathogens infecting various cellular models. One study encapsulated gentamicin in chitosan nanoparticles decorated with phosphatidylcholine to evaluate its efficacy against *L. monocytogenes* and *S. aureus.* The results demonstrated that encapsulation significantly reduced the bacterial load, as measured in CFUs, compared to free gentamicin.^46^ Similarly, another study encapsulated a β-lactam antibiotic, amoxicillin, in combination with potassium clavulanate within mesoporous iron carboxylate-based nanoMOFs (MIL-100(Fe)) to target *S. aureus* infecting macrophage cells. This approach reported a marked reduction in bacterial counts using the antibiotic-loaded nanoMOFs compared to the free antibiotic, highlighting the potential of nanotechnology to enhance intracellular antibiotic efficacy.^47^

While conventional antibiotics such as aminoglycosides, rifamycins, and fluoroquinolones remain the cornerstone of antibacterial therapies, the reviewed studies highlight an increasing interest in nonconventional antibacterial agents, including natural compounds and antibacterial peptides. These agents, often derived from phenolic compounds or bioactive peptides, offer promising alternatives to multidrug-resistant pathogens. For instance, Pashizeh et al. (2024) encapsulated gingerol in alginate-coated niosome nanoparticles to target *S. aureus* and *P. aeruginosa* in a breast cancer cell model. Their findings demonstrated that gingerol encapsulated in alginate-coated niosomes was more effective compared to gingerol encapsulated in uncoated niosomes or free gingerol.^48^ Similarly, Zou et al. (2023) employed glabridin-loaded cinnamaldehyde-dextran nanoparticles to combat MRSA in a macrophage infection model. This system, designed to release its cargo in the acidic lysosomal environment, showed greater efficacy compared to free glabridin.^49^ Phenolic compounds have gained attention for their antibacterial activity, especially against multidrug-resistant pathogens. By disrupting membranes and inhibiting key processes, they offer a promising alternative in combating antimicrobial resistance.^50,51^

The incorporation of novel antibacterial agents into nanoparticle systems not only enhances their efficacy but also addresses critical limitations of conventional therapies such as poor intracellular penetration and systemic toxicity. These findings, along with increased interest in natural products and bioactive compounds, underscore the potential of nanoparticle technologies to overcome barriers posed by intracellular infections and multidrug resistance.

Although all of the studies reviewed report improved antibacterial efficacy through nanoparticle-based delivery, it is not possible to determine which nanoparticle system is superior. This is due to the high heterogeneity in nanoparticle types, formulations, physicochemical properties, and biological models used as well as the variability in how efficacy outcomes are measured and reported. However, key takeaway points from this systematic review include the importance of optimizing nanoparticle size and surface charge and the incorporation of active targeting strategies to enhance intracellular uptake and delivery. Additionally, the choice of cellular and bacterial models is critical to evaluating the therapeutic performance and should be aligned with the pathogen’s intracellular niche and the host’s endocytic pathways. Standardizing infection assay methodologies and reporting practices is essential to moving the field forward. Future research should therefore prioritize the use of *in vivo* models, the exploration of underrepresented pathogens, and the rational design of nanoparticle platforms tailored to the biological context of infection.

## Conclusion

Treating intracellular bacterial infections remains a critical challenge due to antibiotic resistance and immune system evasion strategies employed by these pathogens. Nanotechnology, through the use of nanoparticles to encapsulate antibacterial agents, offers a promising alternative to enhance intracellular penetration and targeted drug delivery, increasing the therapeutic efficacy. However, the lack of comprehensive research on nanoparticulate systems for intracellular infections and the limited use of *in vivo* models highlight significant gaps in current research. It is critical to continue exploring and optimizing these technologies to adapt them to the specific characteristics of each type of bacterial infection, which could open new opportunities to combat resistant infections. Furthermore, standardization of experimental approaches and increased research into underrepresented pathogens are key steps in advancing this field and improving therapeutic outcomes.

## Supporting Information Available

Table S1: Experimental conditions reported for the invasion (gentamicin protection) assay extracted from the methods sections of the included studies (PDF)
