# Supplementary material for: Nanoparticles in Antibacterial Therapy: A Systematic Review of Enhanced Efficacy against Intracellular Bacteria
Source: ACS Omega. 2025 Apr 25;10(17):17070–86. doi: 10.1021/acsomega.5c01813 (PMC12059930; doi:10.1021/acsomega.5c01813)
Supplement: Supplementary file 1 — ao5c01813_si_001.pdf [file ao5c01813_si_001.pdf]

# Nanoparticles in Antibacterial Therapy: A Systematic Review of Enhanced Efficacy Against Intracellular Bacteria

Pablo Mendez-Pfeiffer[1,2*], Manuel G. Ballesteros Monrreal[2], Mayra Mendez-Encinas[2], Dora Valencia[2], Bryan Ortiz[3], Oscar González-Davis[1], Ruben D. Cadena-Nava[1*]

[1] Departamento de Bionanotecnología, Centro de Nanociencias y Nanotecnología, Universidad Nacional Autónoma de México, Km 107 carretera Tijuana-Ensenada, Ensenada Baja California 22860, México.

[2] Departamento de Ciencias Químico Biológicas y Agropecuarias, Universidad de Sonora, campus Caborca, Caborca 83600, Sonora, México.

[3] Instituto de Investigaciones en Microbiología, Facultad de Ciencias, Universidad Nacional Autónoma de Honduras, Tegucigalpa 11101, Honduras.

*Corresponding authors: pablo.mendez@unison.mx; rcadena@ens.cnyn.unam.mx

**Table S1.** Experimental conditions reported for the invasion (gentamicin protection) assay extracted from the methods sections of the included studies.

| BACTERIAL MODEL | TYPE OF CELL | NUMBER OF SEEDED CELLS | MOI | INCUBATION TIME OF BACTERIA WITH CELLS | REMOVAL OF EXTRACELLULAR BACTERIA | INCUBATION TIME WITH NPs TREATMENT | DOI | Ref. |
|---|---|---|---|---|---|---|---|---|
| *Staphylococcus aureus* | RAW 264.7 Macrophages | Not specified | Not specified | 1h | Lysozyme (100 µg/mL) | 0.5 h | 10.1039/d4tb00409d | 52 |
| *Staphylococcus aureus* | RAW264.7 Macrophages | 1x10$^5$ | 100 | 1h | Gentamicin (50 µg/mL) | 2 h | 10.1016/j.biomaterials.2023.122344 | 53 |
| *Staphylococcus aureus* | Macrophages | Not specified | Not specified | 24 h | Lysostaphin (50 µg/mL) | 2 h | 10.1016/j.jconrel.2022.02.030 | 54 |
| *Staphylococcus aureus and Pseudomonas aeruginosa* | HeLa cells | 7x10$^4$ | 100 | 1 h | Gentamicin (100 µg/mL) | 2 h | 10.1016/j.ejpb.2014.03.003 | 55 |
| *Enterococcus faecalis* | HBLAK cells | 2x10$^5$ | 10 | Overnight | Gentamicin/Vancomycin (150/10 µg/mL) | 2 h | 10.1016/j.jconrel.2020.08.048 | 56 |
| *Staphylococcus aureus* | RAW264.7 Macrophages | 1x10$^6$ | 20 | 1 h | Gentamicin (200 µg/mL) - 2 h | 6 h | 10.1016/j.nantod.2023.102137 | 57 |
| *Chlamydia trachomatis* | HeLa cells | 4x10$^4$ | 1 | 1 h | Not specified | 48 h | 10.3390/pharmaceutics14010036 | 58 |
| *Salmonella typhi* | Huh-7 cells | 1x10$^5$ | 100 | 2 h | Only PBS washing | Not specified | 10.1021/acsabm.9b00724 | 59 |
| *Staphylococcus aureus and Salmonella* | RAW264.7 Macrophages | 1x10$^5$ | 10 to 20 | Not specified | Gentamicin (50 µg/mL) | 6 h | 10.1039/c9nr01284b | 33 |
| *Staphylococcus aureus, Pseudomonas aeruginosa* | MDA-MB-231 cells | 2x10$^5$ | 10 | 2 h | Gentamicin (50 µg/mL) - 1 h | 24 h | https://doi.org/10.1016/j.ijbiomac.2023.128957 | 48 |
| *Salmonella typhi* | RAW264.7 Macrophages | Not specified | Not specified | 4 h | 1 % antibiotics | Not specified | 10.1016/j.ijbiomac.2023.124741 | 60 |
| *Chlamydia trachomatis* | RAW264.7 Macrophages | Not specified | 100 | 2 h | Not specified | Not specified | 10.1021/acsinfecdis.3c00131 | 61 |
| *Staphylococcus aureus, Salmonella typhimurium and Enterococcus* | RAW264.7 Macrophages | 5x10$^5$ | 20 | 1 h | Vancomycin (50 µg/mL) - 1.5 h | 4-6 h | 10.1016/j.jconrel.2024.09.028 | 62 |
| *Escherichia coli, Staphylococcus aureus and Mycobacterium marinum* | RAW264.7 Macrophages | 1x10$^6$ | 4 | 6 h | Only PBS washing | 24 h | 10.1016/j.ajps.2024.100926 | 63 |
| *Staphylococcus aureus* | J774A.1 Macrophages | 5x10$^5$ | 5 | 1 h | Gentamicin (1 µg/mL) - 0.5 h | 24 h | 10.1016/j.jconrel.2024.08.029 | 64 |
| *Staphylococcus aureus* | J774-1 Macrophages | 2.5x10$^5$ | 10 | 2 h | Gentamicin - 1, 6 or 24 h | 2 h | 10.1002/ppsc.201800360 | 47 |
| *Staphylococcus epidermidis and Escherichia coli* | RAW264.7 Macrophages and HC-11 cells | Not specified* | Not specified* | 2 h | Not specified* | 4 h | https://doi.org/10.1016/j.xphs.2024.11.006 | 65 |

| | | | | | | | | |
|---|---|---|---|---|---|---|---|---|
| *Staphylococcus aureus* | RAW264.7 Macrophages | Not specified | 100 | 2 h | Gentamicin (100 µg/mL) - 1 h | 12 h | https://doi.org/10.2147/IJN.S476471 | 66 |
| *Klebsiella pneumoniae and Staphylococcus aureus* | RAW264.7 Macrophages and THP-11 Macrophages | $3x10^5$ | 100 | 1 h | Gentamicin (100 µg/mL) - 45 min | Not specified | 10.1016/j.ijpharm.2024.124567 | 67 |
| *Staphylococcus aureus* | RAW264.7 Macrophages | $2x10^4$ | 30 | 0.5 h | Gentamicin (30 µg/mL) | 24 h | 10.1002/adfm.202204906 | 68 |
| *Staphylococcus aureus* | RAW 264.7 Macrophages | Not specified | Not specified | Not specified | Not specified | 12, 24 and 48 h | 10.1016/j.actbio.2023.08.004 | 49 |
| *Staphylococcus aureus* | RAW 264.7 Macrophages | $1x10^5$ | 20 | 2 h | Gentamicin (50 µg/mL) - 1 h | 6 h | 10.1002/anie.202306427 | 69 |
| *Staphylococcus aureus* | RAW 264.7 Macrophages | $1x10^5$ | 10 | 1 h | Only PBS washing | 4 h | 10.3390/pharmaceutics15041037 | 70 |
| *Staphylococcus aureus, Pseudomonas aeruginosa* | RAW 264.7 Macrophages, A549 cells | $3x10^5$ | 100 (P. aeruginosa) - 10 (S. aureus) | 2 h | Gentamicin (200 µg/mL)/ Lysostaphin (10 µg/mL) - 1 h | 4 h | 10.1016/j.ijpharm.2023.122927 | 71 |
| *Staphylococcus aureus* | RAR 264.7 Macrophages | $5x10^4$ | 10 to 20 | 2 h | Gentamicin (50 µg/mL) | 24 h | 10.1016/j.jconrel.2023.03.053 | 72 |
| *Mycobacterium tuberculosis (MTB-H37Ra)* | THP-1 Macrophages | $5x10^4$ | 100 | Not specified | Only PBS washing | Not specified | 10.1021/acsami.3c00101 | 73 |
| *Staphylococcus aureus* | RAW 264.7 Macrophages | $5x10^4$ | 25 | 1 h | Gentamicin (50 µg/mL) - 1 h | 12, 24 and 48 h | 10.1039/d3tb00125c. | 74 |
| *Klebsiella pneumoniae* | MLE12 mouse lung epithelial cell | Not specified | Not specified | 1 h | Gentamicin (100 µg/mL) - 45 min | Not specified | https://doi.org/10.1016/j.nano.2022.102640 | 34 |
| *Helicobacter pylori* | RAW 264.7 Macrophages and GES-1 cells | Not specified | 10 | 2 h | Gentamicin (200 µg/mL) - 2 h | Not specified | https://doi.org/10.1016/j.jconrel.2022.05.044 | 75 |
| *Staphylococcus aureus* | THP-1 Macrophages | Not specified | 10 | 2 h | Gentamicin (50 µg/mL) - 2 h | 12, 24 and 48 h | | 76 |
| *Staphylococcus aureus* | RAW 264.7 Macrophages | $2x10^5$ | 100 | 1 h | Lysostaphin (4 µg/mL) - 0.5 h | 24 h | https://doi.org/10.1016/j.biomaterials.2021.121309 | 77 |
| *Staphylococcus aureus* | RAW 264.7 Macrophages | 7 | Not specified | 1 h | Lysostaphin (200 µg/mL) - 0.5 h | 12 h | https://doi.org/10.1021/acs.biomac.4c00339 | 78 |
| *Klebsiella pneumoniae* | J774A.1 Macrophages | $1x10^5$ | 100 | 1 h | Gentamicin (100 µg/mL) - 0.5 h | 12 h | https://doi.org/10.1016/j.jconrel.2021.11.037 | 79 |
| *Burkholderia thailandensis* | RAW 264.7 Macrophages | Not specified | Not specified | Not specified | Not specified | 3 or 21 h | https://doi.org/10.1021/acsnano.1c05309 | 80 |
| *Staphylococcus aureus* | J774A.1 Macrophages | $2x10^4$ | 10 to 20 | 0.5 h | Gentamicin (30 µg/mL) | 24 h | 10.1039/d1tb01702k | 81 |
| *Mycobacterium bovis BCG* | THP-1 Macrophages | $2.5x10^5$ | 1 | 3 h | Amikacin (200 ng/mL) - 1 h | 20 h | https://doi.org/10.1021/acs.biomac.1c00554 | 82 |

| | | | | | | | | |
|---|---|---|---|---|---|---|---|---|
| *Mycobacterium tuberculosis* | Primary human macrophages | $1x10^5$ | 5 | 2 h | Only PBS washing | 72 h | 10.1002/adhm.202100453 | 83 |
| *Staphylococcus aureus, Listeria monocytogenes, Salmonella typhimurium and Shigella flexneri* | J774A.1 Macrophages | $1x10^5$ | 10/10/10/100 | 1 h | Gentamicin (100 µg/mL) - 1 h | 12 h | https://doi.org/10.1016/j.biomaterials.2020.120344 | 84 |
| *Staphylococcus aureus, Mycobacterium abscessus* | THP-1 Macrophages | $2x10^5$ | 5 and 1 | 3 h | Only PBS washing | 24/72 h | 10.1021/acsinfecdis.4c00045 | 85 |
| *Escherichia coli (EB1-1)* | RAW 264.7 Macrophages | $1x10^5$ | 10 | 1 h | Not specified | 8 h | 10.1039/D0NR01366H | 86 |
| *Staphylococcus aureus* | Caco-2 cells | $1x10^5$ | 10 | 1 h | Only PBS washing | 4 h | 10.3390/nano10040815 | 87 |
| *Listeria monocytogenes and Pseudomonas aeruginosa* | RAW264.7 Macrophages | Not specified | 2 | 1 h | Gentamicin (50 µg/mL) -1 h | 8 h | 10.1016/j.ijbiomac.2020.04.090 | 46 |
| *Staphylococcus aureus* | RAW 264.7 Macrophages | $1x10^4$ | 50 | 0.5 h | Gentamicin (50 µg/mL) - overnight | 6 and 24 h | https://doi.org/10.1016/j.jconrel.2020.03.037 | 88 |
| *Staphylococcus aureus* | RAW 264.7 Macrophages | $1x10^5$ | 10 | 1 h | Only PBS washing | 4 h | https://dx.doi.org/10.1021/acsami.9b22991 | 89 |
| *Staphylococcus aureus* | THP-1 Macrophages and HEK 293 cells | $5x10^5$ | 5 | 2 h | Only PBS washing | 24 h | 10.1016/j.ijpharm.2019.118881 | 90 |
| *Staphylococcus aureus* | A549 lung cells | $1x10^5$ | 10 | 2 h | Gentamicin (50 µg/mL) - 2 h | 1 h | 10.1021/acsabm.8b00821 | 91 |
| *Brucella melitensis and Brucella abortus* | J774A.1 Macrophages | $5x10^5$ | 100 | 1 h | Gentamicin (50 µg/mL) | 18 h | 10.31661/gmj.v8i0.1296 | 92 |
| *Staphylococcus aureus* | HEK 293 kidney cells | $5x10^4$ | Not specified | 2 h | only medium washing | 22 h | 10.1021/acs.molpharmaceut.9b00713 | 93 |
| *Salmonella enterica* | Hep-2 and Caco-2 | Not specified | 100 | 1 h | Gentamicin (50 µg/mL) - 2 h | 2 to 4 h | https://doi.org/10.1002/adhm.201900564 | 94 |
| *Mycobacterium smegmatis* | J774A.1 Macrophages | $1x10^5$ | 20 | 1 h | Not specified | Not specified | https://doi.org/10.1016/j.heliyon.2019.e01539 | 95 |
| *Staphylococcus aureus* | RAW 264.7 Macrophages | $1x10^4$ | 10 | 1 h | Only PBS washing | 4 h | 10.3390/antibiotics8020039 | 96 |
| *Staphylococcus aureus* | HaCaT keratinocytes cell | $1.2x10^5$ | 83 | 3 h | Gentamicin (200 µg/mL) - 3 h | 3 h | 10.3390/polym10050521 | 97 |
| *Salmonella typhimurium and Staphylococcus aureus* | RAW 264.7 Macrophages | Not specified | 2 | 1 h | Gentamicin (50 µg/mL) - 1 h | 12 h | 10.1016/j.carbpol.2019.01.068 | 98 |
| *Mycobacterium tuberculosis* | J774A.1 Macrophages | $1x10^6$ | 10 | 4 h | Not specified | 0, 48 and 72 h | 10.1080/21691401.2018.1559180 | 99 |
| *Mycobacterium tuberculosis* | THP-1 Macrophages, Calu-3 cells | Not specified | 10 | 4 h | Amikacin (200 µg/mL) - 0.5 h | 4 days | https://doi.org/10.1016/j.jconrel.2024.03.013 | 100 |
| *Brucella melitensis* | RAW264.7 Macrophages | Not specified | 10 | 2 h | Only PBS washing | Not specified | 10.1016/j.jconrel.2018.12.024 | 101 |

| | | | | | | | | |
|---|---|---|---|---|---|---|---|---|
| *Shigella flexneri* | Hep-2 cells | Not specified | 25 | 2 h | Gentamicin (50 µg/mL) - 2 h | 3 h | https://doi.org/10.1007/s11095-018-2521-3 | 102 |
| *Klebsiella pneumoniae* | MH-S Macrophages and THP-1 cells | $3x10^5$ | 100 | 1 h | Gentamicin (100 µg/mL) - 45 min | Not specified | 10.1016/j.jconrel.2018.04.040. | 103 |
| *Staphylococcus aureus* | HaCaT keratinocytes cell | $1.5x10^5$ | 100 | 3 h | Gentamicin (X2 MIC) - 1 h | 2 or 5 h | 10.1002/adhm.201701483 | 104 |
| *Staphylococcus aureus* | RAW 264.7 Macrophages | Not specified | Not specified | Not specified | Not specified | Not specified | 10.1002/adhm.202303671 | 105 |
| *Salmonella typhi* | HeLa cells | Not specified | 200 | 2 h | Antibiotic-Antimycotic (X2) | 3.5 h | 10.1038/s41598-017-06014-4 | 106 |
| *Pseudomonas aeruginosa and Listeria monocytogenes* | RAW264.7 Macrophages | Not specified | 10 | 1 h | Gentamicin (50 µg/mL) - 1 h | 2 h | 10.1038/srep18877 | 107 |
| *Yersinia pseudotuberculosis* | Hep-2 cells | Not specified | 25 | 1 h | Gentamicin (50 µg/mL) - 1 h | 2 h | 10.1016/j.jconrel.2015.10.052 | 108 |
| *Francisella tularensis* | THP-1 Macrophages | $1x10^5$ | 10 | 1.5 h | Only PBS washing | 2 and 24 h | 10.1021/acsnano.5b04306 | 109 |
| *Staphylococcus aureus* | RAW 264.7 macrophages, NIH/3T3 cells | $1x10^5$ | 5 | 2 h | Gentamicin (100 µg/mL) - 2.5 h | 20 h | 10.1039/d3tb02430j | 110 |
| *Mycobacterium tuberculosis* | THP-1 Macrophages | Not specified | Not specified | 1 h | Gentamicin (100 µg/mL) - 45 min | Not specified | 10.1002/smll.201500937 | 111 |
| *Staphylococcus aureus* | MC3T3-E1 preosteoblastic cell | $6x10^4$ | Not specified | 2 h | Only PBS washing | 1 to 48 h | 10.1016/j.msec.2014.01.008 | 112 |

*Experimental conditions could not be retrieved from supplementary material; MOI: Multiplicity of Infection. NPs: Nanoparticles.
